# Deletions and de novo mutations of *SOX11* are associated with a neurodevelopmental disorder with features of Coffin–Siris syndrome

**DOI:** 10.1136/jmedgenet-2015-103393

**Published:** 2015-11-05

**Authors:** Annmarie Hempel, Alistair T Pagnamenta, Moira Blyth, Sahar Mansour, Vivienne McConnell, Ikuyo Kou, Shiro Ikegawa, Yoshinori Tsurusaki, Naomichi Matsumoto, Adriana Lo-Castro, Ghislaine Plessis, Beate Albrecht, Agatino Battaglia, Jenny C Taylor, Malcolm F Howard, David Keays, Aman Singh Sohal, DDD collaboration, Susanne J Kühl, Usha Kini, Alisdair McNeill

**Affiliations:** 1 Institute for Biochemistry and Molecular Biology, Ulm University, Ulm, Germany; 2 National Institute for Health Research Biomedical Research Centre, Wellcome Trust Centre for Human Genetics, University of Oxford, Oxford, UK; 3 Department of Clinical Genetics, Chapel Allerton Hospital, Leeds, UK; 4 Department of Clinical Genetics, St George's Hospital, London, UK; 5 Department of Genetic Medicine, Floor A, Belfast City Hospital, Belfast, UK; 6 Laboratory of Bone and Joint Diseases, Center for Integrative Medical Sciences, RIKEN, Tokyo, Japan; 7 Department of Human Genetics, Yokohama City University Graduate School of Medicine, Yokohama, Japan; 8 Department of Neuroscience, Pediatric Neurology Unit, Tor Vergata University of Rome, Rome, Italy; 9 Service de génétique, CHU de Caen—Hôpital de la Côte de Nacre, Caen, France; 10 Institut fur Humangenetik, Universitatsklinikum Essen, Universitat Duisburg-Essen, Essen, Germany; 11 The Stella Maris Clinical Research Institute for Child and Adolescent Neurology and Psychiatry, Pisa, Italy; 12 Institute of Molecular Pathology, Vienna, Austria; 13 Paediatric Neurology, Birmingham Children's Hospital, Birmingham, UK; 14 Department of Clinical Genetics, Oxford University Hospitals NHS Trust, Oxford, UK; 15 INSIGNEO Institute for in silico medicine, Sheffield University, Sheffield, UK; 16 Sheffield Institute for Translational Neuroscience, Sheffield University, Sheffield, UK; 17 Sheffield Clinical Genetics Service, Sheffield Children's Hospital, Sheffield, UK

**Keywords:** Developmental, Clinical genetics, Copy-number, Diagnostics tests

## Abstract

**Background:**

*SOX11* is a transcription factor proposed to play a role in brain development. The relevance of *SOX11* to human developmental disorders was suggested by a recent report of *SOX11* mutations in two patients with Coffin–Siris syndrome. Here we further investigate the role of *SOX11* variants in neurodevelopmental disorders.

**Methods:**

We used array based comparative genomic hybridisation and trio exome sequencing to identify children with intellectual disability who have deletions or de novo point mutations disrupting *SOX11*. The pathogenicity of the *SOX11* mutations was assessed using an in vitro *gene* expression reporter system. Loss-of-function experiments were performed in xenopus by knockdown of Sox11 expression.

**Results:**

We identified seven individuals with chromosome 2p25 deletions involving *SOX11.* Trio exome sequencing identified three de novo *SOX11* variants, two missense (p.K50N; p.P120H) and one nonsense (p.C29*). The biological consequences of the missense mutations were assessed using an in vitro gene expression system. These individuals had microcephaly, developmental delay and shared dysmorphic features compatible with mild Coffin–Siris syndrome. To further investigate the function of *SOX11*, we knocked down the orthologous gene in xenopus. Morphants had significant reduction in head size compared with controls. This suggests that *SOX11* loss of function can be associated with microcephaly.

**Conclusions:**

We thus propose that *SOX11* deletion or mutation can present with a Coffin–Siris phenotype.

## Introduction

The SOX proteins are transcription factors with a shared motif called the SRY box, a high mobility group (HMG) DNA binding domain. The SOX proteins regulate gene expression, acting as either transcriptional activators or repressors, in multiple tissues, and so play crucial roles in multiple developmental processes.^1^ *SOX11* is thought to play a crucial role in brain development. In humans, neuron production begins on embryonic day 42.^2^ In the fetus, the neuronal progenitors are located in the subventricular zone. After production in the subventricular zone, neurons migrate outwards into the cortical layers and undergo differentiation into mature neurons. The linked processes of neuronal production from progenitor cells and differentiation into functioning neurons must be tightly regulated to ensure proper brain development.^2^ *SOX11* null mice have reduced cortical neurogenesis secondary to reduced proliferation and abnormal differentiation of neuronal progenitor cells.^3^ This results in *SOX11* null mice having reduced brain weights and thin cerebral cortices.^3^ There is also evidence that *SOX11* plays a role in ocular development. *Sox11* knockdown in zebrafish induces microphthalmia with or without iris coloboma.^4^ *SOX11* represents a strong candidate gene for human neurodevelopmental disease.

Haploinsufficiency of other *SOX* genes is associated with human disease. Mutations in *SOX10* are associated with Waardenburg–Hirschprung disease,^5^ *SOX9* mutations with campomelic dysplasia^6^ and haploinsufficiency of *SOX5* is reported to cause intellectual disability.^7^ Tsurusaki *et al*^8^ reported two children with Coffin–Siris syndrome (CSS, OMIM#135900) and de novo mutations in *SOX11.* CSS is characterised by developmental delay/intellectual disability, feeding difficulties, facial dysmorphology, microcephaly and hypoplastic nails of the fifth digits.^9^ Both of the mutations reported by Tsurusaki *et al*^8^ were within the HMG domain and interfered with the ability of *SOX11* to induce gene transcription in vitro. This implicates regulation of gene expression as a mechanism by which *SOX11* contributes to human brain development. Multiple genes regulated at a transcriptional level by *SOX11* have been identified.^10^ *SOX11* can also repress transcription of genes important for neurodevelopment. In *SOX11* null mice, *LIS1* was upregulated significantly.^3^ Altered levels of *SOX11* thus have the potential to cause dysregulation of multiple genetic pathways, with clear potential to disrupt developmental processes.

Here we report seven individuals with chromosome 2p25 deletions including *SOX11* and three with de novo *SOX11* mutations. These individuals presented with a phenotype that had some clinical features of CSS, but not a classical phenotype that would readily permit clinical diagnosis of CSS. An in silico analysis demonstrated that expression of *SOX11* is highest in the brain during early fetal life, suggesting a role for *SOX11* in human neurodevelopment. Knockdown of Sox11 in xenopus laevis was associated with microcephaly in the morphants.

## Materials and methods

### Ascertainment of *SOX11* deletion (2p25.2 deletions) and mutation cases

Individuals with deletion of chromosome 2p25.2, which included the *SOX11* gene, were identified through the DECIPHER collaboration. Deletions were confirmed by FISH. None of the deletions identified were present in the recently published CNV map of the human genome, which integrates CNV data from healthy individuals from multiple data sets such as the database of genomic variants.^11^ Two individuals with *SOX11* mutations were identified in the deciphering developmental disorders (DDD) study (data freeze of 1133 children). DDD methodology has been described.^12^ A third individual with a *SOX11* mutation was identified by exome sequencing via the Genetics of Structural Brain Abnormalities and Learning Disabilities Study (Wales Research Ethics Committee 12/WA/0001).^13^ Mutations were confirmed by Sanger sequencing.

### In silico assessment of pathogenicity of novel *SOX11* mutations

The predicted effect of the *SOX11* missense variants was examined using SIFT, PolyPhen and the ‘Have Your Protein Explained’ tool (http://www.cmbi.ru.nl/hope/home). Evolutionary conservation of mutated amino acids was assessed by aligning orthologues in Ensembl (http://www.ensembl.org/index.html). The presence of *SOX11* variants in normal control populations was queried using the ExAC browser (http://exac.broadinstitute.org/gene/ENSG00000176887).

### Cell transfection and luciferase reporter assays

The *SOX11* open-reading frame clone was purchased from Promega (Tokyo, Japan) and *SOX11* mutants (c. 150G>C; p. Lys50Asn and c.359C>A; p. Pro120His) generated by site-directed mutagenesis with the KOD-Plus-Mutagenesis Kit (Toyobo, Osaka, Japan). Wildtype (WT) and mutant *SOX11* cDNAs were PCR amplified and cloned into the p3xFLAG-CMV-14 mammalian expression vector (Sigma, St Louis, Missouri, USA). The *GDF5* promoter 5′-flanking sequence (−448/+319) was PCR amplified and cloned into the pGL3-basic vector (Promega). All constructs were verified by Sanger sequencing. Human *SOX11* cDNA can be obtained from GenBank/EMBL/DDBJ nucleotide core database under the accession code AB028641.1. Transfection and luciferase reporter assays were performed as previously described.^8^

### In silico assessment of *SOX11* expression in developing brain

Variation of *SOX11* expression levels in the human brain over different developmental stages was investigated using RNA-sequencing data from the Brainspan atlas of the developing human brain (http://www.brainspan.org/). Methods are described in the online supplementary methods.

### Sox11 knockdown in xenopus laevis embryos

*Xenopus laevis* embryos were obtained and cultured according to standard protocols and staged as described previously.^14–16^ All morpholino oligonucleotides (MOs) were obtained by GeneTools, LLC, OR, USA, and resuspended in Diethylpyrocarbonate (DEPC)-treated water. For loss-of-function experiments, Sox11 MO (30 ng per blastomere) was injected.^14^ For control experiments, the standard control MO suggested by GeneTools was used. MOs were injected bilaterally into both dorso-animal blastomeres of *Xenopus* embryos at eight-cell stage to target anterior neural tissue. As a lineage tracer, 0.5 ng *gfp* RNA was co-injected in all experiments to ensure proper injections. For cephalic evaluations, *Xenopus* embryos at stage 45 were fixed with formaldehyde and imaged using a Zeiss Axiophot microscope. Head area and interpupillary distance in knockdown and control morphants was compared using the Mann–Whitney U test (GraphPad prism).

## Results

### Clinical case reports

#### Individuals with *SOX11* deletions

**Figure 1:**
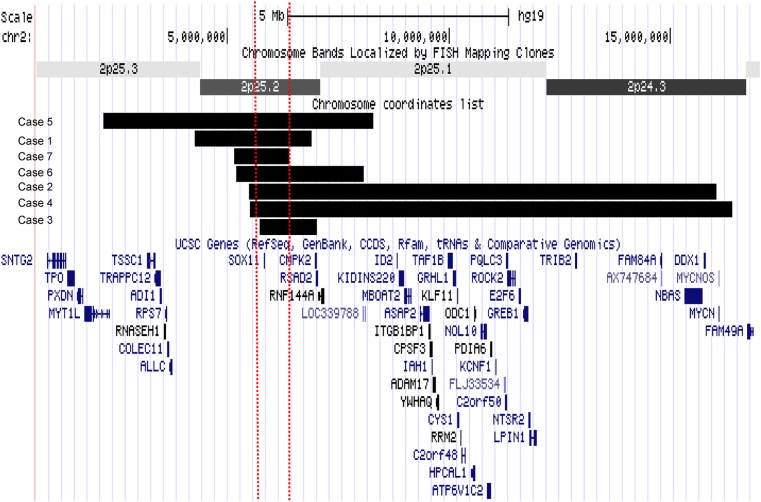
Schematic diagram of deletions. The deletions are displayed as a custom track in UCSC genome browser with refseq genes using hg 19. The horizontal black bars represent the deletion in each patient and the two dashed vertical lines represent the smallest region of overlap. The smallest region of overlap contains only the *SOX11* gene.

**Figure 2:**
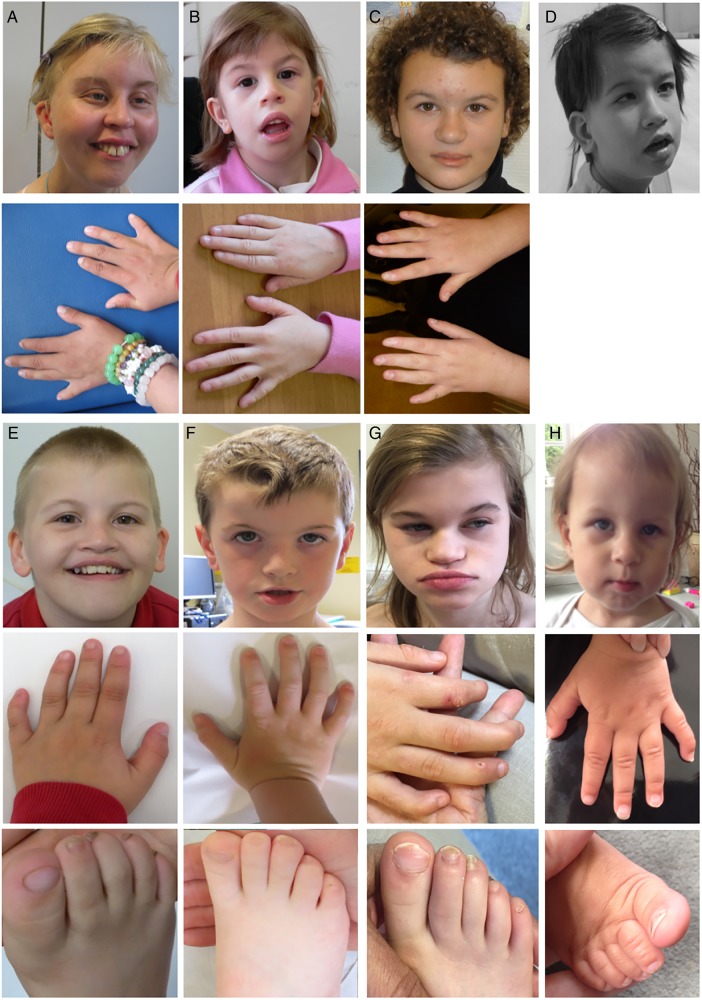
Clinical photographs of study participants. (A). Case 4: facial photograph in top panel, photograph of hands in lower panel (note fifth finger clinodactyly). (B). Case 2: facial photograph in top panel, photograph of hands in lower panel (note fifth finger clinodactyly). (C) Case 3: facial photograph in top panel, photograph of hands in lower panel (note fifth finger clinodactyly). (D) Case 1: facial photograph in top panel. Reproduced from Lo-Castro *et al*.^17^ (E) Boy with c.150G>C (p. Lys50Asn) *SOX11* mutation (case 9). Facial photograph in top panel, hand in middle panel (note fifth finger clinodactyly) and foot in lowermost panel (note broad hallux and 2–3 toe syndactyly). (F) Boy with c.87C>A (p. Cys29*) *SOX11* mutation (case 10). Facial photograph in top panel, hand in middle panel (note fifth finger clinodactyly) and foot in lowermost panel (note broad hallux, 2–3 toe syndactyly and hypoplasia of nail of fifth toe). (G). Girl with c.359C>A (p. Pro120His) *SOX11* mutation (case 8). Facial photograph in top panel, hand in middle panel (note fifth finger clinodactyly) and foot in lowermost panel (note broad hallux and hypoplasia of nail of fifth toe). (H) Case 7. Facial photograph in top panel, photograph of hands in lower panel (note fifth finger clinodactyly and small nails on fifth finger).

Clinical details, deletion mapping and photographs are given in table 1 and figures 1 and 2. Case 1 is a 12-year-old girl, the first child of healthy non-consanguineous parents (previously described at 7 years old^17^). She was born at 41 weeks of gestation by caesarean section due to fetal bradycardia and oligohydramnios. Birth weight was 3685 g (50th centile), length 50 cm (50th centile) and birth head circumference 35 cm (50th centile). At the age of 12 years, her head circumference was 50.4 cm (<3rd centile), height 152 cm (25–50th) and weight 44 kg (75th centile). She walked without support at 18 months and has poor fine motor skills. She started speaking single words at the age of 3 years and 6 months and has had no further language development. She manifests repetitive and stereotyped movements with hyperactivity and autism. She had a non-verbal IQ of 54. She was generally well apart from gastro-oesophageal reflux. On examination she has mild facial asymmetry with right microphthalmia and a wide mouth with thick lips and large, simplified ears. Bilateral fifth finger clinodactyly, cutaneous syndactyly of toes 2–3, scoliosis and inverted nipples were also noted. CGH was reported as arr 2p25(4291420–6905655)(hg 19)x1 with deletion of the *SOX11* gene only. The deletion was not present in either parent.

**Table 1:** Summary of demographic, genetic and clinical characteristics

	Case 1	Case 2	Case 3	Case 4	Case 5	Case 6
Demographics (sex, age in years)	Female 12 years	Female 5 years, 11 months	Female 14 years	Female 25 years	Male 34 years	Female 13 years
Genetic result	2p25(4291420–6905655)(hg 19)x1	2p25(5511851–16027633)x1	2p25(5838893–7023548)(hg19)x1	2p25(5535091–16398225)x1	2p25(2231163–300707)x1	2p25(5209876–8078809)x1
Growth	Height 152 cm (25–50th) Weight 44 kg (75th) OFC 50.4 cm (<3rd)	Height 102.5 cm (<2nd) Weight 14.5 kg (<2nd) OFC 47 cm (<2nd)	Height 158 cm (50th) OFC 52 cm (2nd)	Height 162 cm (25th) Weight 50 kg (10th) OFC 49 cm (<0.4th)	Height 150 cm (3rd) OFC 53 cm (3rd)	Microcephalic
Development	Walking—18 months Speech—single words	Walking—22 months Speech—18 months	Walking—20 months Speech—no delay	Severe intellectual disability	Intellectual disability	Speech—delayed
Feeding	GORD	GORD	Poor feeding as a neonate	Gastritis		Poor feeding as a neonate
Neurological	Autism			Febrile seizures	Epilepsy Autism	Epilepsy
Skeletal system	5th finger clinodactyly 2–3 toe syndactyly scoliosis	5th finger clinodactyly	5th finger clinodactyly 2–3 toe syndactyly scoliosis	5th finger clinodactyly 2–3 toe syndactyly scoliosis	Scoliosis	5th finger clinodactyly
Eye findings	Rt microophthalmia	Hypermetropia		Hypermetropia		
Other	Inverted nipples		Inverted nipples			

	Case 7	Case 8	Case 9	Case 10
Demographics (sex, age in years)	Female 2 years, 3 months	Female 12 years, 6 months	Male 11 years old	Male 6 years, 10 months
Genetic test	2p25(5166592–6400126)x1	c.359C>A, p. Pro120His	c.150G>C, p. Lys50Asn	c.87C>A, p. Cys29* Nonsense
Growth	Weight 10 kg (2nd) OFC 47.5 cm (50th)	Height 89.4 cm (0.4th) Weight 12.15 kg (0.4th) OFC 46.5 cm (0.4th)	Height 109.4 cm (9th) Weight 18.45 kg (9th) OFC 48.2 cm (0.4th)	Height 124.5 cm (75th) Weight 29 kg (91st) OFC 54 cm (75th)
Development	Walking—15 months Speech—single words	Walking—30 months Speech—none	Walking—48 months Speech—36 months	Walking—16 months Speech—significant delay
Feeding	Poor feeding as a neonate	Poor feeding as a neonate	Poor feeding as a neonate	
Neurological		Autism Absence seizures		
Skeletal system	5th finger clinodactyly Hypoplasia nail 5th toe	5th finger clinodactyly Hypoplasia nail 5th toe	5th finger clinodactyly 2–3 toe syndactyly Hypoplasia nail 5th toe	5th finger clinodactyly 2–3 toe syndactyly Hypoplasia nail 5th toe
Eye findings		Oculomotor apraxia	Hypermetropia	Oculomotor apraxia
Other				

Case 2 is a 5-year 11-month-old girl, the first child of healthy non-consanguineous parents. She was born at 36 weeks of gestation by caesarean section because of intrauterine growth restriction. Birth weight was 1850 g (<10th centile) and head circumference 31 cm (<10th centile). She first walked independently at 22 months old and spoke her first words at 18 months. At 5 years and 11 months old, weight was 14.5 kg (<3rd centile), height 102.5 cm (<3rd centile) and OFC 47 cm (<2nd centile). She had troublesome gastro-oesophageal reflux and a high degree hypermetropia. On examination she had sparse hair, epicanthic folds and a wide mouth with thick lips. Bilateral clinodactyly of the fourth and fifth fingers with generalised joint laxity was noted. Brain MRI showed agenesis of the posterior third of the corpus callosum. CGH was reported as arr 2p25(5511851–16027633**)**x1(hg 19). The deletion encompassed the *SOX11 and MYCN* genes. The deletion was not present in either parent.

Case 3 is a 14-year-old girl, the first child of healthy non-consanguineous parents. She was born at 37 weeks of gestation. Birth weight was 2840 g (9th centile) and head circumference 33 cm (2nd centile). She had a weak suck and difficulty feeding in the neonatal period. She first walked at the age of 20 months. Her fine motor skills were impaired. She had no speech delay. At 14 years old, her height was 158 cm (50th) and head circumference was 52 cm (<2nd). She was dysmorphic with a high forehead, narrow palpebral fissures, a smooth philtrum, micrognathia and a high arched palate. A scoliosis, inverted nipples, bilateral fifth finger clinodactyly and 2–3 toe syndactyly were noted. CGH was reported as arr 2p25(5838893–7023548)(hg 19)x1. The deletion included *SOX11, RSAD2* and *CMPK2* genes. The deletion was not found in the mother. The father was not available for testing.

Case 4 is a 25-year-old woman, the third child of healthy non-consanguineous parents. Pregnancy was complicated by reduced fetal movements. Birth weight was 3130 g (25th centile). Choanal stenosis and persistent ductus arteriosus were present in the neonatal period. She first walked at 4 years and spoke at 3 years old. At the age of 25 years old, her height was 162 cm (25th centile), weight 50 kg (10th centile) and head circumference 49 cm (<0.4th centile). She has severe intellectual disability and aggressive behaviour. She is physically well but has hypermetropia. She has a flat face with upslanting palpebral fissures and wide mouth with thick lips. Short second fingers with bilateral fifth finger clinodactyly was present along with skin syndactyly of toes 2–3 and 4–5. CGH was reported as arr 2p25(5535091–16398225)(hg 19)x1 with deletion of *SOX11* and *MYCN*. The deletion was not found in either parent.

Case 5 is a 34-year-old man, the third child of healthy non-consanguineous parents (patient 16 from the study of De Rocker *et al*^18^). He was born at term. Birth weight was 3750 g (75th). He had intellectual disability, speech delay and epilepsy. On examination at age 34 years, height was 150 cm (3rd centile) and head circumference 53 cm (3rd centile, corrected for height). He had facial dysmorphism: microretrognathia, short philtrum and mild trigonocephaly. Scoliosis and cryptorchidism were also noted. CGH was reported as arr 2p25(2231163–8300707)(hg 19)x1 with deletion of the *SOX11* and *MYT1L* genes. This was paternally inherited; a detailed phenotypic description of the father was not available, but he was reported to be epileptic and had intellectual disability and speech delay.^18^

Case 6 is a 13-year-old girl, ascertained via DECIPHER with limited information. She was born at term. She presented as an infant with feeding difficulties, microcephaly and seizures. CGH was reported as arr2p25(5209876–8078809)(hg 19)x1 with deletion of *SOX11, RSAD2, CMPK2* and *RNF144A*. It was not possible to test parental samples.

Case 7 is a 2-year-old girl, the first child of healthy non-consanguineous parents. She was born at 39 weeks of gestation by elective caesarean section. Birth weight was 3.5 kg (50th). There were feeding difficulties and poor weight gain in the first month of life. This resulted in hospital admission. Gastro-oesophageal reflux was diagnosed and the feeding difficulties resolved with gaviscon. At this time generalised hypotonia was noted. There was a history of mild gross motor delay; she first sat independently at 10 months and walked at 15 months. At the age of 2 years, she had begun to run. There were no concerns relating to fine motor skills. There was mild speech delay; at the age of 2 years, she was not producing two word phrases. She was physically well. On examination her head circumference was 47 cm (50th centile) and weight 10 kg (2nd centile). She had deep set eyes, full cheeks and thick lips. There was sparse scalp hair. She had bilateral fifth finger clinodactyly and hypoplasia of the nails of the fifth fingers and toes. CGH was reported as arr2p25.2(5166592–6400126)(hg19)x1. Only the *SOX11* gene was deleted, and it was not possible to test parental samples.

### Individuals with *SOX11* mutations

Case 8 is a 12-year and 6-month-old girl, the first child of healthy non-consanguineous parents. She was born at 41 weeks of gestation. Birth weight was 3118 g (26th centile). There was poor feeding in the neonatal period requiring nasogastric feeding. She had global developmental delay; first walking at 2 years and 6 months old. She has never spoken. At the age of 12 years and 6 months, her height was 89.4 cm (<0.4th), weight 12.15 kg (<0.4th) and head circumference 46.5 cm (<0.4th). She has profound intellectual disability, severe autism, absence seizures, bruxism and a tendency to pick at her skin. On examination facial dysmorphism (sunken eyes, depressed nasomaxillary area and wide mouth with thick lips), bilateral fifth finger clinodactyly and hypoplasia of the toe nails of her little toes were noted. She had oculomotor apraxia. Brain MRI was normal. CGH was normal. Exome sequencing identified a de novo, heterozygous missense variant in SOX11 (c.359C>A, p. Pro120His; NM_003108.3).

Case 9 is an 11-year-old boy, the first child of healthy non-consanguineous parents. Pregnancy was complicated by third trimester bleeding. He was born at 40 weeks of gestation. Birth weight was 3500 g (50th). He was readmitted to hospital at 3 weeks of age with poor feeding (immature suck, choking and regurgitation of bottle feeds) and failure to thrive. He had global developmental delay; first walking at age 2 years and speaking his first words at age 3. At 11 years of age, he was described as being aggressive with poor attention and no sense of danger. He spoke only in short sentences and was in a special needs school. On examination he was on the 9th centile for height and weight and had a head circumference under the 0.4^th^ centile. He had bilateral conductive deafness, cryptorchidism, hypermetropia and squint. He had malar flattening, a short philtrum and tented upper lip and a left preauricular skin tag. Bilateral 2–3 cutaneous toe syndactyly and fifth finger clinodactyly was also noted. CGH was normal. Exome sequencing identified a de novo, missense variant in *SOX11* (c.150G>C, p. Lys50Asn).

Case 10 is a 6-year-old boy, the child of healthy non-consanguineous parents. He was born by planned caesarean section with a birth weight of 4.08 kg (75th). He presented with mild global developmental delay; first walking at 16 months of age and having significantly delayed expressive speech. He attends a learning unit and has over-excitable behaviour. He is generally well apart from enuresis. He was 124.5 cm tall (75th centile), 29 kg (91st centile) and head circumference was 54 cm (75th centile). On examination he was noted to have slightly coarse facial features with wide mouth and thick lips, a prominent metopic suture, arched well-defined eyebrows and midline anterior hairline upsweep. He had oculomotor apraxia. Bilateral fifth finger clinodactyly and fifth fingernail hypoplasia was noted along with bilateral fourth/fifth toenail hypoplasia. Radiographs of the hands and feet were normal. A brain MRI detected hypoplasia of the inferior aspect of the cerebellar vermis. Array CGH (180k, Agilent) was normal. Exome sequencing identified a de novo, nonsense variant in *SOX11* (c.87C>A, p. Cys29*).

### In silico analysis of *SOX11* missense variants

**Figure 3:**
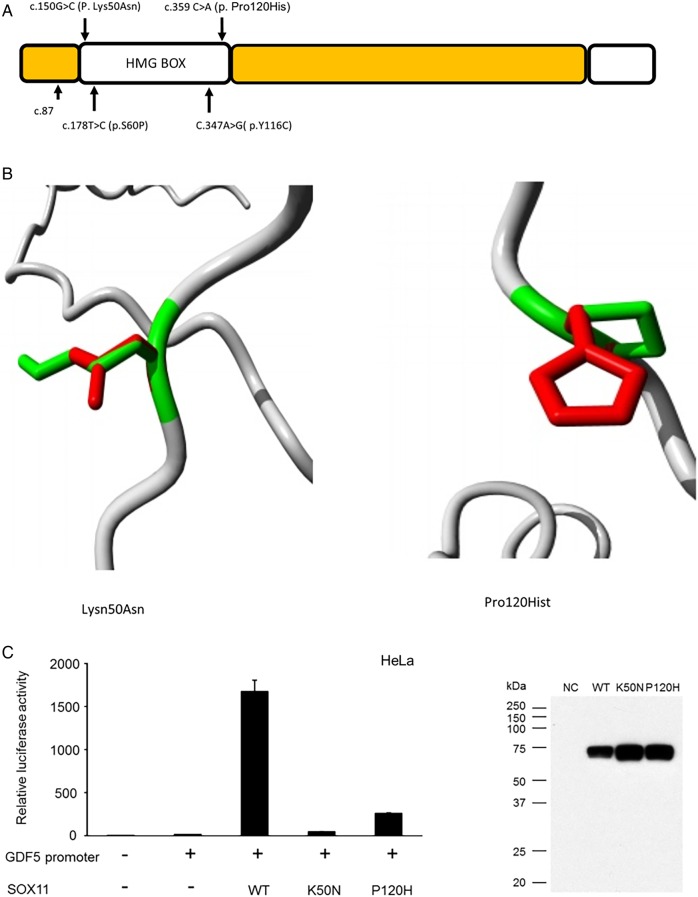
SOX11 variants identified in the current study. (A) Schematic diagram of *SOX11* protein demonstrating location of three reported sequence variants. The p.S60P and p.Y116C variants reported by Tsurusaki *et al*^8^  are also shown, the structural effects of these mutations can be found in reference 8. (B and D) Models demonstrating alteration of SOX11 protein structure associated with the two missense variants. Green areas represent the wildtype residue while the red area indicates the structure adopted by the mutant amino acid. Both the missense variants were in the DNA binding domain of *SOX11* and predicted to alter its structure, thus interfering with DNA binding. (C) Bar chart demonstrating that the two*SOX11* missense variants had reduce ability to activate the GDF5 promoter in an in vitro reporter system. The adjacent western blot confirms that the mutant proteins were stably expressed during the experiment.

The c.150G>C (p. Lys50Asn) variant was predicted by SIFT to be deleterious (score of 0) and PolyPhen to be probably damaging (score of 1). The c.359C>A (p. Pro120His) variant was predicted by SIFT to be deleterious (score of 0) and by PolyPhen to be probably damaging (score of 0.996). Both mutations localise to the HMG (DNA binding) domain (figure 3). The ‘Have Your Protein Explained’ tool identified that in the c.359C>A (p. Pro120His) variant, the mutant residue (histidine) is larger and more hydrophilic than the WT amino acid. This was predicted to interfere with DNA binding and protein–protein interaction. In the c.150G>C (p. Lys50Asn) variant, asparagine is noted to be of smaller size and neutral charge compared with the WT amino acid. This was also predicted to interfere with DNA binding. Both variants are found at evolutionary conserved amino acids. Neither variant was found in the exAC database, nor was the c.87C>A, p. Cys29* mutation.

### In vitro assessment of effect of *SOX11* mutations on transcriptional activity

Luciferase reporter assays in HeLa cells indicate that both the p.Lys50Asn and p.Pro120His variants display reduced ability to activate the GDF5 promoter compared with WT protein (figure 3).

#### Knockdown of Sox11 in xenopus laevis

**Figure 4:**
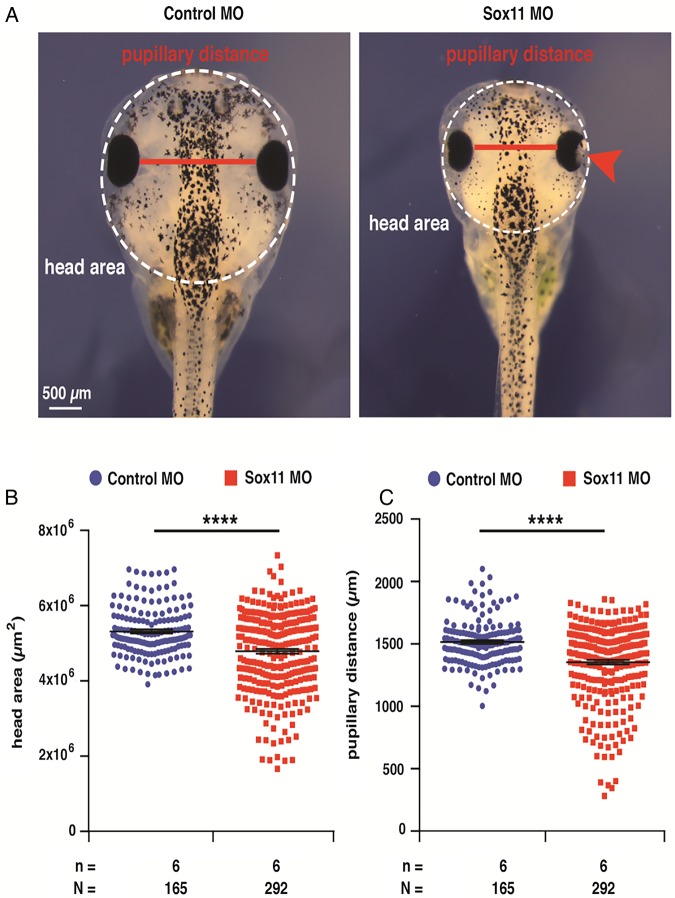
Sox11 knockdown leads to microcephaly in *Xenopus laevis*. (A) Bilateral injection of Sox11 MO results in significant smaller heads measured by the head area (white dotted circles) and the pupillary distance (red lines) compared with bilateral control MO injections. In addition, Sox11 morphants show an eye phenotype as previously described (red arrowhead; Cizelsky *et al*^14^). (B) Statistical evaluation of the measured head area. (C) Statistical evaluation of the measured pupillary distance. N, number of individual embryos analysed. ****, p≤0.0001. p Values were calculated by a non-parametric Mann–Whitney rank sum test.

Knockdown of Sox11 by MO injection resulted in a significant reduction in head area and interpupillary distance compared with controls (both p<0.0001 on Mann–Whitney U test). Figure 4 shows a representative morphant and control. There was no increased death rate among the morphants and the controls.

### Expression of *SOX11* in human brain

**Figure 5:**
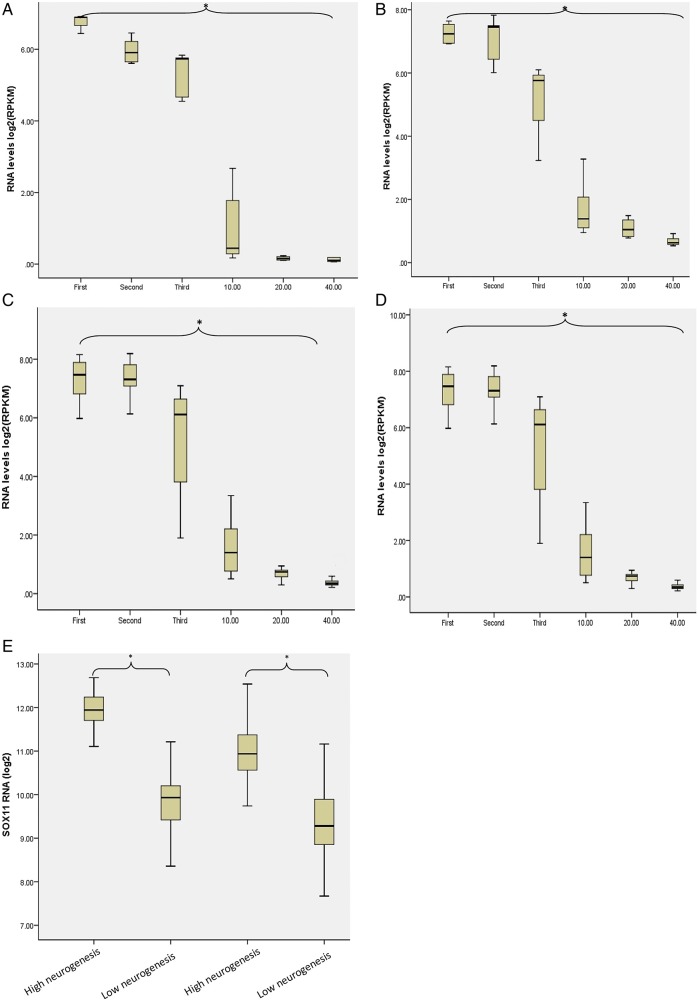
Expression of SOX11 in developing human brain. (A–D) Changes in SOX11 expression levels as measured by RNA-sequencing in the cerebellum, hippocampus, prefrontal cortex and striatum, respectively. Columns labelled first, second and third refer to trimesters of pregnancy. Column 10 represents the first decade of life, 20 the second decade of life and 40 the third and fourth decades. There was a significant decline in SOX11 expression levels with increasing age as assessed by the Kruskal–Wallis test (p<0.01). (E) Microarray data demonstrating that SOX11 is expression is higher in brain regions with high levels of neurogenesis (periventricular) compared with areas with low levels of neurogenesis (cerebellum, thalamus, brain stem), *p<0.01. The first two columns represent 15 weeks of gestation while the second two columns represent 21 weeks of gestation.

For all brain regions examined, *SOX11* expression levels (RNA-seq) were highest in the first trimester of pregnancy and then fell significantly (Kruskal–Wallis, p<0.01) to reach a nadir in the fourth decade of life (figure 5A–D). *SOX11* expression levels (microarray) were significantly greater in brain areas with high levels of neurogenesis compared with areas of low neurogenesis (Mann–Whitney U test p<0.01) (figure 5E) at both 15 weeks and 21 weeks of gestation.

## Discussion

Here we report seven patients with chromosome 2p25 deletions, which include *SOX11,* and three individuals with de novo *SOX11* point mutations. The individuals with deletions had several phenotypic features in common (table 1). Microcephaly was reported in all but two. All had developmental delay, with particularly marked speech delay. A shared facial dysmorphology of wide mouth and thick lips could be discerned. One of the patients reported here had trigonocephaly, and so it is interesting to note that a 2004 report describes a boy with trigonocephaly, cleft palate and multiple minor anomalies with deletion of *SOX11* resulting from an unbalanced translocation 46,XY,t(2;17)(p25;q24).^19^ Fifth finger clinodactyly and cutaneous syndactyly of toes 2–3 was a frequent examination finding. There was considerable variability between deletion cases—likely reflecting the different CNV sizes. The individuals with de novo *SOX11* mutations had several shared phenotypic features. Microcephaly, low birth weight and neonatal feeding difficulties associated with hypotonia were frequent findings. Developmental delay was reported in all cases apart from patient 2 in Tsurusaki *et al*'s^8^ report. Hypoplasia of the distal phalanx of the fifth finger, broad halluces, 2–3 toe syndactyly and hypoplasia of the nail of the fifth toe are also frequent. Ocular abnormalities were present in both deletion and mutation cases; case 1 had right microphthalmia, cases 2, 4 and 7 had hypermetropia and squint while cases 7 and 9 had oculomotor apraxia. Our report confirms that *SOX11* mutations and deletions can be associated with a neurodevelopmental disorder, which manifests features of CSS.

The clinical features associated with *SOX11* deletion or mutation overlap with other syndromes. Hypoplasia of the fifth fingers with dysmorphism and intellectual disability can be observed in mosaic trisomy 9,^20^ deafness-onychodystrophy–osteodystrophy–mental retardation syndrome^21^ and phenytoin embryopathy.^22^ There is overlap between mild forms of Cornelia de Lange syndrome and individuals with *SOX11* mutations.^23^ It is important to note that none of our cohort had a clinical diagnosis of CSS prior to CGH or exome sequencing being performed. In retrospect, the individuals we report had clinical features compatible with CSS (eg, hypoplasia of fifth finger) but did not present with classical dysmorphic features of CSS, which would enable a clinical diagnosis to be readily made. This is analogous to the presentation of *ARID1B* mutations. *ARID1B* mutations have been identified as the most common cause of CSS and also in children with intellectual disability who have subtle features of CSS, but who would not have been diagnosed with this syndrome on the basis of their phenotype alone.^24^ However, we suggest that certain features of CSS, such as the pattern of hair distribution (synophrys, sparse scalp hair but increased body hair) and fifth finger hypoplasia in combination, should lead to CSS being included in differential diagnoses for children with a neurodevelopmental disorder.

Several other genes within the deleted 2p25 regions could contribute to the observed phenotypes. In the centromeric deletions (cases 2 and 4), it is highly likely that *MYCN* deletion contributes to the phenotype. Deletions and mutations of *MYCN* are associated with Feingold syndrome.^25^ The classical features of Feingold syndrome are microcephaly, intestinal atresias and brachymesophalangy of the second and fifth fingers. The severe intellectual disability reported in case 4 is unusual for individuals with Feingold syndrome.^25^ In the individual with a telomeric deletion (case 5), loss of *MYTL1* is likely to contribute to the phenotype. However, case 5 has borderline microcephaly, while the other individuals in the report of De Rocker *et al*^18^ who had similar deletions involving *MYT1L* but not *SOX11* tended to have macrocephaly. This suggests that *SOX11* haploinsufficiency may exert a powerful, negative influence on brain growth. Cases 1 and 7 have no genes other than *SOX11* in the deleted region, while the deletion in cases 3 and 6 also contains the *CMPK2*, *RSAD2* and *RNF144A* genes. *CMPK2* encodes a mitochondrial nucleoside monophosphate kinase,^26^ *RSAD2* encodes viperin, which is an antiviral protein,^27^ and *RNF144A* is an E3 ubiquitin ligase involved in DNA damage repair and apotosis.^28^ Haploinsufficiency scores indicate that heterozygous loss of these genes is unlikely to cause a neurodevelopmental disorder. This provides evidence that *SOX11* deletion alone can be associated with a neurodevelopmental phenotype.

The fact that both heterozygous deletions and mutations of *SOX11* are associated with microcephaly suggests that loss of function and haploinsufficiency may be the underlying mechanism. The two missense variants we describe are within the HMG DNA binding domain (as were the two previously reported missense variants^8^) while the c.87 C>A variant would be predicted to lead to premature termination of translation prior to this domain. *SOX11* is a single exon gene. Nonsense-mediated decay (NMD) may not occur with mutations in the final exon of a gene,^29^ so it is possible that a mutant *SOX11* transcript may not undergo NMD. However, the c.87C>A mutation occurs before the HMG domain in *SOX11*, so it is likely that any protein product will be unable to bind DNA and induce gene expression. The mutant protein is also likely to be unstable given its truncated nature. It is thus likely that all three mutations will interfere with the ability of *SOX11* to regulate its target genes. Our luciferase reporter gene assays provide further evidence in support of this as they indicate a reduced ability of mutant *SOX11* to induce gene expression. An in vitro study of *SOX11* overexpressing cells identified multiple genes upregulated by *SOX11*, which are relevant to neurogenesis and brain development.^10^ We hypothesise that haploinsufficiency of *SOX11* could potentially reduce expression of these target genes at critical points in brain development, resulting in a neurodevelopmental disorder.

The expression pattern of *SOX11* in the human brain is in keeping with the gene playing a role in neurogenesis during embryonic development. We show that *SOX11* expression peaks in the first three months of in utero life and declines thereafter. Since neurogenesis in the fetal brain is largely completed by mid-gestation,^2^ this temporal expression pattern fits with *SOX11* being involved in neurogenesis in the fetal brain. The spatial expression pattern of *SOX11* in fetal brain also suggests that *SOX11* is involved in neurogenesis since *SOX11* expression was significantly higher in the ventricular zone than in areas with relatively low levels of neurogenesis. That two of our cases had brain malformations provides further evidence that *SOX11* functions in human neurodevelopment.

Data from animal models also suggests that *SOX11* plays an important role in eye development since *SOX11* knockdown in zebrafish can cause ocular malformations.^4^ In addition, variants in *SOX11* have also been identified in two individuals with iris coloboma and no neurodevelopmental phenotype.^4^ This may be explained by the fact that the sequence variants in these individuals were not located in the DNA binding HMG domain of SOX11 protein, while variants reported herein associated with CSS were predicted to interfere with DNA binding.

Our experiments in *Xenopus* embryos indicate that loss of Sox11 is associated with microcephaly. We previously demonstrated that Sox11 depletion leads to smaller eyes.^14^ This is in line with our current study as case 1 shows microphthalmia. Moreover, the microophthalmia phenotype can be rescued by co-injection of WT Sox11 RNA.^14^ The fact that (1) Sox11 depletion leads to an eye phenotype^14^ similar to case 1 (our current study), (2) co-injection of Sox11 MO together with WT Sox11 RNA results in a rescue of the Sox11 MO-induced eye phenotype^14^ and (3) Sox11 MO injection does not lead to an increased death rate compared with control MO injection suggests that the microcephaly phenotype of Sox11 knockdown in *X. laevis* is not explained by non-specific toxic effects of MO injection. The precise mechanism by which loss of Sox11 results in microcephaly in *Xenopus*, however, is still unclear. Our previous work on *Xenopus* eye development indicates that Sox11 knockdown does not alter proliferation but is associated with increased neuronal apoptosis.^14^ This suggests that *SOX11* may also function as a neuronal survival factor in brain development.

In conclusion, we describe a series of individuals with *SOX11* deletions or de novo mutations presenting a neurodevelopmental disorder, which had clinical features compatible with CSS. The two *SOX11* missense variants reported here are the only plausibly pathogenic *SOX11* variants identified from over 1000 exomes performed on probands with a developmental disorder in the DDD study.^30^ *SOX11* variants are a rare cause of neurodevelopmental disorders. That both deletions and mutations of *SOX11* can cause CSS is in keeping with data reported for *ARID1A* and *ARID1B,* deletions or truncating mutations of which also cause CSS.^31^ Deletion mapping, in our small cohort, suggests that *SOX11* deletion, although likely pathogenic in itself, can also act as part of a contiguous gene deletion along with loss of *MYT1L* or *MYCN*. The mechanism is likely to be *SOX11* haploinsufficiency with dysregulation of the *SOX11* target genes and consequent disruption of brain development. *SOX11* is itself induced by the BAF (BRG1-associated or HRBM-associated factors) complex, leading to neuronal differentiation.^32^ It is noteworthy that many other genes mutated in CSS function in the BAF complex.^32^ The majority of genes in the BAF complex in which mutations have been identified are associated with a CSS phenotype (eg, ARID1A, ARID1B, SMARCB1, SMARCA4 and SMARCE1), while mutations in SMARCA2 cause Nicolaides–Baraitser syndrome.^31^ Our report thus reinforces the importance of the BAF complex in CSS. In conclusion, the current study provides evidence that *SOX11* is a further member of the SOX protein family associated with human neurodevelopmental disease.

## Supplementary Material

Web supplement

## References

[1] Pillai-Kastoori L, Wen W, Morris AC Keeping an eye on SOXC proteins. Dev Dyn 2015;244:367–76. 10.1002/dvdy.24235 25476579 PMC4344926

[2] Urban N, Guillemot F Neurogenesis in the embryonic and adult brain: same regulators, different roles. Front Cell Neurosci 2014;8:396 10.3389/fncel.2014.00396 25505873 PMC4245909

[3] Wang Y, Lin L, Lai H, Parada LF, Lei L Transcription factor SOX11 is essential for both embryonic and adult neurogenesis. Dev Dyn 2013;242:638–53. 10.1002/dvdy.23962 23483698

[4] Pillai-Kastoori L, Wen W, Wilson SG, Strachan E, Lo-Castro A, Fichera M, Musumeci SA, Lehmann OJ, Morris AC SOX11 is required to maintain proper levels of hedgehog signalling during vertebrate ocular morphogenesis. PLoS Genet 2014;7:e1004491 10.1371/journal.pgen.1004491 PMC4091786 25010521

[5] Pingault V, Bondurand N, Kuhlbrodt K, Goerich DE, Préhu MO, Puliti A, Herbarth B, Hermans-Borgmeyer I, Legius E, Matthijs G, Amiel J, Lyonnet S, Ceccherini I, Romeo G, Smith JC, Read AP, Wegner M, Goossens M SOX10 mutations in patients with Waardenburg-Hirschprung disease. Nat Genet 1998;18:171–3. 10.1038/ng0298-171 9462749

[6] Kowk C, Weller PA, Guioli S, Foster JW, Mansour S, Zuffardi O, Punnett HH, Dominguez-Steglich MA, Brook JD, Young ID Mutations in SOX9 the gene responsible for Campomelic dysplasia and autosomal sex reversal. Am J Hum Genet 1995;57:1028–36. 7485151 PMC1801368

[7] Lamb AN, Rosenfeld JA, Neill NJ, Talkowski ME, Blumenthal I, Girirajan S, Keelean-Fuller D, Fan Z, Pouncey J, Stevens C, Mackay-Loder L, Terespolsky D, Bader PI, Rosenbaum K, Vallee SE, Moeschler JB, Ladda R, Sell S, Martin J, Ryan S, Jones MC, Moran R, Shealy A, Madan-Khetarpal S, McConnell J, Surti U, Delahaye A, Heron-Longe B, Pipiras E, Benzacken B, Passemard S, Verloes A, Isidor B, Le Caignec C, Glew GM, Opheim KE, Descartes M, Eichler EE, Morton CC, Gusella JF, Schultz RA, Ballif BC, Shaffer LG Haploinsufficiency of SOX5 at 12p12.1 is associated with developmental delays with prominent language delay, behavioural problems and mild dysmorphic features*.* Hum Mutat 2012;33:728–40. 10.1002/humu.22037 22290657 PMC3618980

[8] Tsurusaki Y, Koshimizu E, Ohashi H, Phadke S, Kou I, Shiina M, Suzuki T, Okamoto N, Imamura S, Yamashita M, Watanabe S, Yoshiura K, Kodera H, Miyatake S, Nakashima M, Saitsu H, Ogata K, Ikegawa S, Miyake N, Matsumoto N De Novo SOX11 mutations cause Coffin-Siris syndrome. Nat Comm 2014;5:4011. 10.1038/ncomms5011 24886874

[9] Santen GW, Clayton-Smith J, ARID1B-CSS Consortium. The ARID1B phenotype: what we have learned so far. Am J Med Genet C Semin Med Genet 2014;166C:276–89. 10.1002/ajmg.c.31414 25169814

[10] Sha L, Porteus D, Blackwood D, Muir W, Pickard B SOX11 target genes: implications for neurogenesis and psychiatric illness. Acta Neuropsych 2012;24:16–25. 10.1111/j.1601-5215.2011.00583.x 25288455

[11] Zarrei M, MacDonald JR, Merico D, Scherer SW A copy number variation map of the human genome*.* Nat Rev Genet 2015;16:172–83. 10.1038/nrg3871 25645873

[12] Wright CF, Fitzgerald TW, Jones WD, Clayton S, McRae JF, van Kogelenberg M, King DA, Ambridge K, Barrett DM, Bayzetinova T, Bevan AP, Bragin E, Chatzimichali EA, Gribble S, Jones P, Krishnappa N, Mason LE, Miller R, Morley KI, Parthiban V, Prigmore E, Rajan D, Sifrim A, Swaminathan GJ, Tivey AR, Middleton A, Parker M, Carter NP, Barrett JC, Hurles ME, FitzPatrick DR, Firth HV, DDD study. Genetic diagnosis of developmental disorders in the DDD study: a scalable analysis of genome-wide research data. Lancet 2015;385:1305–14. 10.1016/S0140-6736(14)61705-0 25529582 PMC4392068

[13] Pagnamenta AT, Howard MF, Wisniewski E, Popitsch N, Knight SJ, Keays DA, Quaghebeur G, Cox H, Cox P, Balla T, Taylor JC, Kini U Germline recesive mutations in PI4KA are associated with perisylvian polymicrogyria, cerebellar hypoplasia and arthrogryposis. Hum Mol Genet 2015;24:3732–41. 10.1093/hmg/ddv117 25855803 PMC4459391

[14] Cizelsky W, Hemple A, Metzig M, Tao S, Holleman T, Kuhl M, Kuhl SJ SOX4 and SOX11 function during Xenopus laevis eye development. PLoS ONE 2013;8:e69372 10.1371/journal.pone.0069372 23874955 PMC3715537

[15] Sive HL, Grainger RM, Harland RM Early development of Xenopus laevis: a laboratory manual. Cold Spring Harbor Laboratory Press, Cold Spring Harbor, NY, 2000.

[16] Nieuwkoop PD, Faber J Normal table of Xenopus laevis (Daudin): a systematical and chronological survey of the development from the fertilized egg till the end of metamorphosis. Garland Pub New York, 1994.

[17] Lo-Castro A, Giana G, Fichera M, Castiglia L, Grillo L, Musumeci SA, Galasso C, Curatolo P Deletion 2p25.2:a cryptic chromosome abnormality in a patient with autism and mental retardation detected using aCGH*.* Eur J Med Genet 2009;52:67–70. 10.1016/j.ejmg.2008.09.004 18992374

[18] De Rocker N, Vergult S, Koolen D, Jacobs E, Hoischen A, Zeesman S, Bang B, Béna F, Bockaert N, Bongers EM, de Ravel T, Devriendt K, Giglio S, Faivre L, Joss S, Maas S, Marle N, Novara F, Nowaczyk MJ, Peeters H, Polstra A, Roelens F, Rosenberg C, Thevenon J, Tümer Z, Vanhauwaert S, Varvagiannis K, Willaert A, Willemsen M, Willems M, Zuffardi O, Coucke P, Speleman F, Eichler EE, Kleefstra T, Menten B Refinement of the critical 2p25.3 deletion region: the role of MYT1L in intellectual disability and obesity. Genet Med 2014;17:460–6. 10.1038/gim.2014.124 25232846

[19] Czako M, Riegel M, Morava E, Bajnoczky K, Kosztolanyi G Opitz “C” trigonocephaly-like syndrome in a patient with terminal deletion of 2p and partial duplication of 17q. Am J Med Genet A 2004;131:310–12. 10.1002/ajmg.a.30249 15540175

[20] Burns DA, Campbell E Twenty-five additional cases of trisomy 9 mosaic: birth information, medical conditions and developmental status. Am J Med Genet A 2015;167:997–1007. 10.1002/ajmg.a.36977 25755087

[21] Campeau PM, Hennekam RC; DOORS Syndrome Collaborative Group. DOORS syndrome: phenotype, genotype and comparison with Coffin-Siris syndrome. Am J Med Genet C Semin Med Genet 2014;166C:327–32. 10.1002/ajmg.c.31412 25169651

[22] Sabry MA, Farag TI Hand anomalies in fetal-hydantoin syndrome: from nail/phalangeal hypoplasia to unilateral acheiria. Am J Med Genet 1996;62:410–12. 10.1002/ajmg.1320620403 8723073

[23] Boyle MI, Jespersgaard C, Brondum-Neilsen K, Bisgaard AM, Tumer Z Cornelia de Lange syndrome*.* Clin Genet 2014;88:1–12. 10.1111/cge.12499 25209348

[24] Hoyer J, Ekici AB, Endele S, Popp B, Zweier C, Wiesener A, Wohlleber E, Dufke A, Rossier E, Petsch C, Zweier M, Göhring I, Zink AM, Rappold G, Schröck E, Wieczorek D, Riess O, Engels H, Rauch A, Reis A Haploinsufficiency of ARID1B, a member of the SWI/SNF-A chromatin remodelling complex, is a frequent cause of intellectual disability. Am J Hum Genet 2012:90;565–72. 10.1016/j.ajhg.2012.02.007 22405089 PMC3309205

[25] Cognet M, Nougayrede A, Malan V, Callier P, Cretolle C, Faivre L, Genevieve D, Goldenberg A, Heron D, Mercier S, Philip N, Sigaudy S, Verloes A, Sarnacki S, Munnich A, Vekemans M, Lyonnet S, Etchevers H, Amiel J, de Pontual L Dissection of the MYCN locus in Feingold syndrome and isolated oesophageal atresia*.* Eur J Hum Genet 2011;19:602–6. 10.1038/ejhg.2010.225 21224895 PMC3083612

[26] Xu Y, Johansson M, Karlsson A Human UMP-CMP kinase 2, a novel nucleoside monophosphate kinase localized in mitochondria. J Biol Chem 2008;283:1563–71. 10.1074/jbc.M707997200 17999954

[27] Upadhyay AS, Vonderstein K, Pichlmair A, Stehling O, Bennett KL, Dobler G, Guo JT, Superti-Furga G, Lill R, Överby AK, Weber F Viperin is an iron-sulfur protein that inhibits genome synthesis of tick-born encephalitis virus via radical SAM domain activity. Cell Microbiol 2014;16:834–48. 10.1111/cmi.12241 24245804

[28] Ho SR, Mahanic CS, Lee YJ, Lin WC RNF44A an E3 ubiquiting ligase for DNA-PKcs promotes apoptosis during DNA damage. Proc Natl Acad Sci 2014;111:E2646–2655. 10.1073/pnas.1323107111 24979766 PMC4084471

[29] Sulem P, Helgason H, Oddson A, Stefansson H, Gudjonsson SA, Zink F, Hjartarson E, Sigurdsson GT, Jonasdottir A, Jonasdottir A, Sigurdsson A, Magnusson OT, Kong A, Helgason A, Holm H, Thorsteinsdottir U, Masson G, Gudbjartsson DF, Stefansson K Identification of a large set of rare complete human knockouts. Nat Genet 2015;47:448–52. 10.1038/ng.3243 25807282

[30] Deciphering Developmental Disorders Study. Large-scale discovery of novel genetic causes of developmental disorders. Nature 2015;519:223–8. 10.1038/nature14135 25533962 PMC5955210

[31] Sim JC, White SM, Lockhart PJ ARID1B-mediated disorders: mutations and possible mechanisms. Intractable Rare Dis Res 2015;4:17–23. 10.5582/irdr.2014.01021 25674384 PMC4322591

[32] Ninkovic J, Steiner-Mezzadri A, Jawerka M, Akinci U, Masserdotti G, Petricca S, Fischer J, von Holst A, Beckers J, Lie CD, Petrik D, Miller E, Tang J, Wu J, Lefebvre V, Demmers J, Eisch A, Metzger D, Crabtree G, Irmler M, Poot R, Götz M The BAF complex interacts with Pax6 in adult neuronal progenitors to establish a neurogenic cross-regulatory transcriptional network. Cell Stem Cell 2013;4:403–18. 10.1016/j.stem.2013.07.002 PMC4098720 23933087

