# Supplementary material for: Deletions and de novo mutations of SOX11 are associated with a neurodevelopmental disorder with features of Coffin–Siris syndrome
Source: J Med Genet. 2015 Nov 5;53(3):152–62. doi: 10.1136/jmedgenet-2015-103393 (PMC4789813; doi:10.1136/jmedgenet-2015-103393)
Supplement: Web supplement [file jmedgenet-2015-103393-s1.pdf]

ONLINE SUPPLEMENTAL METHODS

In silico assessment of *SOX11* expression in developing brain

Variation of *SOX11* expression levels in the human brain over different developmental stages was investigated by analysing RNA-sequencing data from the Brainspan atlas of the developing human brain (http://www.brainspan.org/). Normalised sample-level RNA-seq data was downloaded and matched to phenotype data for the brain region and age of each sample. The median and interquartile ranges of Log-2 RPKM (reads per kilobase per million) for *SOX11* were calculated for each trimester of pregnancy, age 0-10 years, age 10-20 years and age 30-40 years for each brain region. This was done for the cerebellum, hippocampus, striatum and prefrontal cortex. Alterations in *SOX11* RPKM between developmental stages were sought using the Kruskall-Wallis test (PASW statistics 18, IBM computing).

Variation of *SOX11* expression in different anatomical areas of the fetal brain was examined using microarray data from the Brainspan atlas from a 15 week female fetus and a 21 week female fetus. *SOX11* expression (log2 normalised) was compared between brain areas with high levels of neurogenesis (ventricular zone and medial, lateral and caudal ganglionic eminences) and low levels (thalamus, cerebellum, brain stem) using the Mann-Whitney U-test (PASW statistics 18, IBM computing).
